# SIR Flat CAP (Safety In Radiology - Flat-Packed Compact Airborne Precaution): A Low-Cost, Portable, Negative-Pressure Isolation Barrier Shield for Protecting Frontline Healthcare Workers

**DOI:** 10.7759/cureus.46345

**Published:** 2023-10-02

**Authors:** Shao J Ong, Ian Renfrew, Deborah X Khoo, Denise A Choong, Hui L Koh, Deborah S Ng, Lycia Teo, Joseph K Lee, Linda Yuen, Koon Liang Chia, Priscilla X Chen, Yi Ming Teo, Bertrand Ang, Swee Tian Quek

**Affiliations:** 1 Radiology, National University of Singapore, Singapore, SGP; 2 Radiology, National University Hospital, Singapore, SGP; 3 Interventional Radiology, Royal London Hospital, London, GBR; 4 Anesthesia, National University Hospital, Singapore, SGP; 5 Psychiatry, Ng Teng Fong General Hospital, Singapore, SGP; 6 Radiology, University of North Carolina, Chapel Hill, USA

**Keywords:** radiology safety, ultrasound, negative pressure barrier shield, aerosol-generating procedures, qualitative fit testing, portable barrier shield, barrier shield, covid-19, supine cough generation model

## Abstract

Introduction

Multiple barrier shields have been described since the start of the COVID-19 pandemic. Most of these are bulky and designed for use in the main anesthetic or radiology departments.

We developed a portable, negative-pressure barrier shield designed specifically for portable ultrasound examinations. A novel supine cough generation model was developed together with a reverse qualitative fit test to simulate real-world aerosol droplet generation and dispersion for evaluating the effectiveness of the barrier shield. We report the technical specifications of this design, named “SIR Flat CAP” from Safety In Radiology - Flat-packed Compact Airborne Precaution, as well as its performance in reducing the spread of droplets and aerosols.

Methods

The barrier shield was constructed using 1 mm acrylic panels, clear packing tape, foam double-sided tape, and surgical drapes. Negative pressure was provided via hospital wall suction.

A supine cough generation model was developed to simulate cough droplet dispersal. A reverse qualitative fit test was used to assess for airborne transmission of microdroplets.

Results

The supine cough generation model was able to replicate similar results to previously reported supine human cough generation dispersion. The use of the barrier shield with negative-pressure suction prevented the escape of visible droplets, and no airborne microdroplets were detected by reverse qualitative fit testing from the containment area.

Conclusions

The barrier shield significantly reduces the escape of visible and airborne droplets from the containment area, providing an additional layer of protection to front-line sonographers.

## Introduction

The Coronavirus Disease 2019 (COVID-19) pandemic caused by the SARS-CoV-2 virus has caused unprecedented stress and burnout in healthcare systems worldwide. The variant (B.1.1.529) named Omicron and its emerging subvariants demonstrate a significant increase in transmissibility with reports of probable airborne transmission and are leading a new wave of infection with unprecedented numbers of new cases [1,2].

Associated complications of COVID-19 disease include increased incidence of thrombosis and liver injury from COVID-19 drug therapy [3,4,5]. Doppler ultrasound is often a first-line imaging investigation to evaluate for portal and hepatic venous thrombosis and hepatic arterial flow. Each ultrasound scan requires prolonged close contact between sonographer and patient, with procedure time much longer due to the challenges of scanning severely ill patients in the Intensive Care Units (ICU).

Various barrier shields have been described since the start of the pandemic [6,7]. The International Society for Magnetic Resonance in Medicine (ISMRM) Safety Committee has now also recommended the use of negative-pressure barrier shields in patients who are unable to wear appropriate personal protective equipment (PPE) to help protect healthcare personnel [8].

We developed a novel supine cough generation model and a reverse qualitative fit test to simulate real world aerosol droplet generation and dispersion for evaluating a low-cost, portable, flat-packed, negative-pressure barrier shield for use in the ultrasound examination setting. We report the technical specifications of this design, named “SIR Flat CAP” from Safety In Radiology - Flat-packed Compact Airborne Precaution, as well as its performance in reducing the spread of droplets and aerosols. 

## Materials and methods

Safety In Radiology (SIR) - Flat Compact Airborne Precaution (CAP)

The barrier shield was made using standard off-the-shelf 1 mm clear acrylic panels measuring 30x45 cm from a hobby craft shop, a 2.5 m standard hospital vacuum suction tubing, clear packing tape, foam double-sided tape, and 30 mm surgical roll prep drape (3M™ Steri-Drape™ Roll Prep Drape, 3M, Bracknell, United Kingdom).

**Figure 1:**
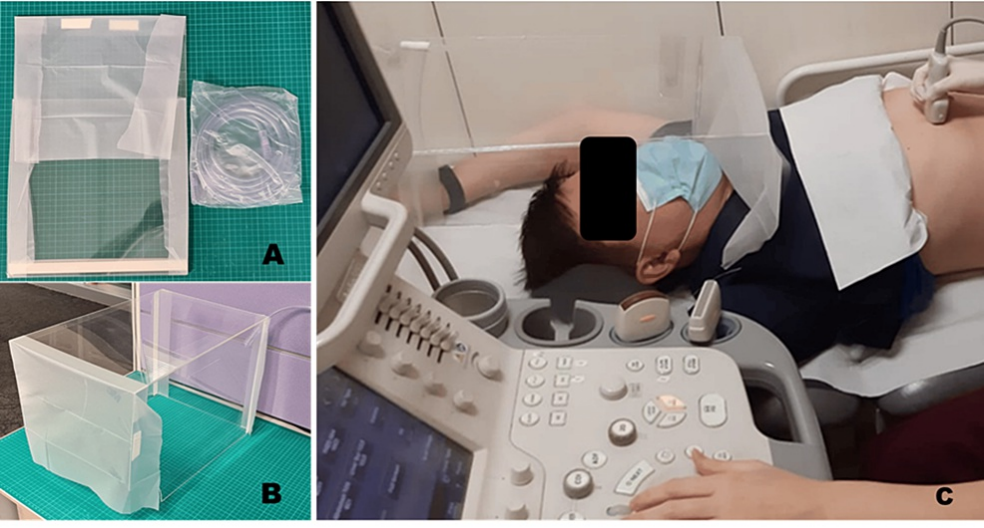
SIR Flat CAP in collapsed flat packed form (A); SIR Flat CAP assembled and ready for use with suction tubing attached via the top right-hand corner (B); Use of SIR Flat CAP in abdominal ultrasound examinations (C)

The top panel was used uncut, the two side panels had a 10 cm radius quarter circle cut out to facilitate patient shoulder position, and the rear panel was cut to 30x30 cm with a 1 cm cut in the top right-hand corner to facilitate suction tubing access to provide a negative-pressure environment. The panels were spaced 5 mm apart and secured with clear packing tape on both sides. The rear of the side panel was lined with foam double-sided tape along its length while the front with the shoulder cut out had 5 cm segments of foam double-sided tape in the middle. Steri-Drape was then attached across the front of the top panel and extended 5cm onto either side. Steri-Drape was attached to the rear panel on the entire length of both sides and cut to 5 cm for overlap. The barrier shield was then folded flat for storage and transport (Figure 1A) and can be rapidly assembled for use (Figure 1B), with the connection of the suction tubing to wall suction. The barrier shield would then be placed over the head of the patient with the Steri-Drape forming a curtain over the thorax when in use (Figure 1C). 

Supine cough generation model

Disposable draw sheets were laid out and fixed onto a flat surface. Sheets were visually checked, ensuring no contaminants were present.

**Figure 2:**
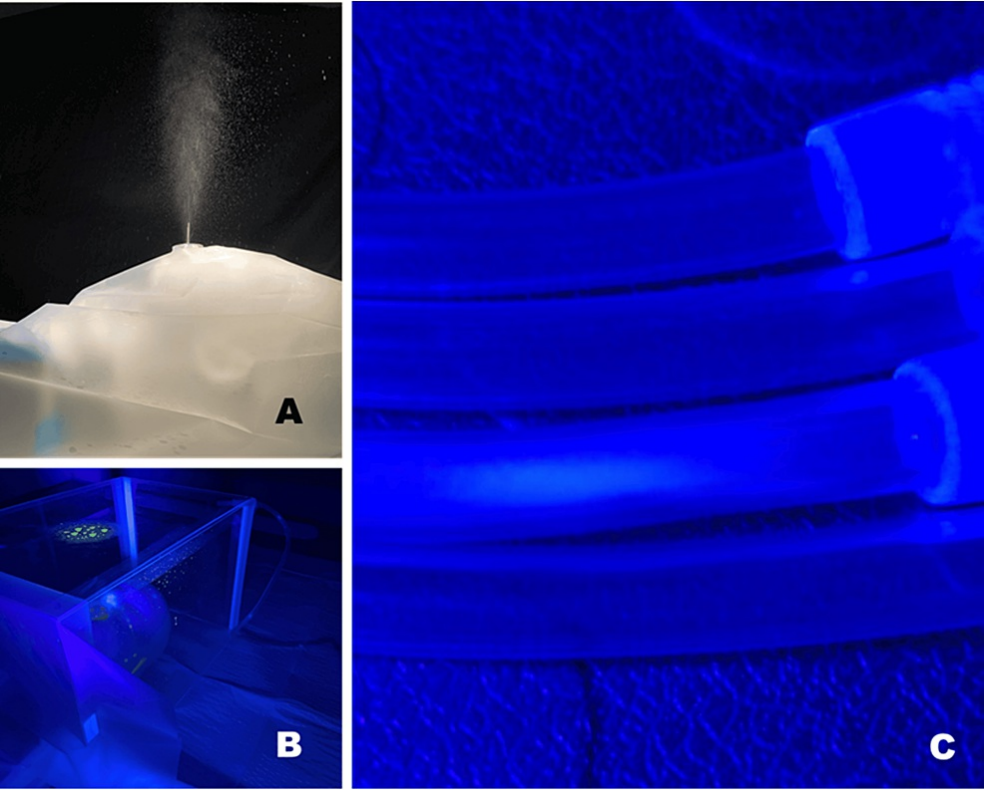
Aerosol generation in our supine cough generation model taken with a high-speed camera (A); Fluorescent-simulated aerosolized cough droplets are contained within the confines of SIR Flat CAP (B); top and bottom tubes are control, the second tube from the top is the wall vacuum end of the suction tubing, and the third tube is the SIR Flat CAP end of the suction tubing with faint fluorescence from de-posited microdroplets (C)

A Hudson RCI Micro Mist nebulizer mask cup (Teleflex Medical, North Carolina, USA) was attached to the mouth end of a 7ft long oxygen tubing and channeled through the simulated airway of a manikin. The nebulizer mask cup was filled with 10 mL of water-soluble non-toxic indicator solution (Illuminisafe, Department of Diagnostic Imaging, National University Hospital, Singapore). At the distal end, a 60 mL Luer lock syringe attached to a gate valve (Floswitch, Merit Medical, Galway, Ireland) was used to generate varying gas pressure to approximate the force of a sneeze or cough [9]. Room air was compressed to varying degrees to create between 1.25 room ATM (standard atmospheric pressure) and 2 ATM. The release of the Floswitch locking mechanism allowed for the sudden transmission of pressure via the oxygen tubing into the Micro Mist mask cup to simulate human cough droplet generation (Figure 2). Each simulated cough was repeated three times before measurement.

Negative-pressure droplet and aerosol barrier shield model

SIR Flat CAP was set up over the cough generation model. Cough simulations were repeated with and without continuous suction through the suction tubing attached to a Medi-Vac Flex Advantage Flexible Liners fluid and bacteria trap (Cardinal Health, Ohio, USA) before venting via standard hospital wall suction.

Reverse qualitative fit test

Volunteers among the study team members were subjected to a negative COVID-19 antigen lateral flow test (Panbio, Abbott, Maidenhead, UK) within six hours preceding the experiment. An aerosol-generating qualitative fit apparatus (3M™ Qualitative Fit Test Apparatus FT-30, 3M, Bracknell, United Kingdom) was set up in the middle of a deployed barrier shield at the expected location of the patient's head. The study member was then positioned unmasked, with their head at the distal front edge of the barrier shield to simulate the relative position of the patient's head and the operator’s head when leaning over to perform an ultrasound of the spleen or left kidney. Five puffs of the qualitative fit test apparatus were performed, and the subject was requested to breathe deeply for two minutes. Simulations were performed with the subjects blinded to the experimental content setup. Normal saline was used as a negative control. The effect of suction was controlled with suction tubing connected or disconnected to reduce subject bias due to audible changes from switching off the wall vacuum.

Optical clarity

Optical clarity after repeated cleaning with ammonia-based Mikrozid Sensitive wipes (Schulke, Norderstedt, Germany), Virusolve+® Sporicidal Wipes (Amity International, Barnsley, UK), and 70% isopropyl alcohol wipes were performed with off-cut panels from the construction as previously described. The light transmission test was carried out using a calibrated light detector (RaySafe Xi, Unfors RaySafe AB, Sweden).

COVID-19 viral antigen deposition

Informed written consent was obtained from all volunteer development team members. All volunteers were provided with the identical suction tubing to that used in SIR Flat CAP throughout the length of the study. As per hospital protocol, all COVID-19-positive members of staff were required to self-isolate at home to prevent viral spread to patients and the general public.

Volunteer study team members who had tested positive for COVID-19 infection on self-isolation at home with mild symptoms participated in the experiment within 24 hours of the first positive test. Each member utilized their suction tubing identical to that used in SIR Flat CAP and was required to breathe normally completely through the tubing for 10 minutes. After 10 minutes, the inner lumen of the suction tubing at the proximal end was swabbed and evaluated with a COVID-19 antigen lateral flow test kit (Panbio, Abbott, Maidenhead, UK). A simultaneous COVID-19 antigen lateral flow test was also performed on the member using a mid-turbinate swabbing technique.

## Results

Reverse qualitative fit test

Without SIR Flat CAP, all nine assessors were able to clearly identify the bitter-tasting fit test solution. With the use of SIR Flat CAP but without the suction, two out of nine assessors reported a faint taste of the test solution. None of the assessors were able to taste the bitterness with the barrier shield and the wall suction at the maximal flow rate. No false positive rates were reported with saline use by all nine assessors (Table 1).

**Table 1:** Individual results from the reverse qualitative fit test using the FT30 bitter-tasting aerosol solution

Assessor	Barrier shield and saline with suction	Barrier shield and FT30 with suction	Barrier shield and saline without suction	Barrier shield and FT30 without suction	Saline without barrier shield	FT30 without barrier shield
1	Negative	Negative	Negative	Negative	Negative	Positive
2	Negative	Negative	Negative	Negative	Negative	Positive
3	Negative	Negative	Negative	Mild positive	Negative	Positive
4	Negative	Negative	Negative	Negative	Negative	Positive
5	Negative	Negative	Negative	Negative	Negative	Positive
6	Negative	Negative	Negative	Negative	Negative	Positive
7	Negative	Negative	Negative	Negative	Negative	Positive
8	Negative	Negative	Negative	Negative	Negative	Positive
9	Negative	Negative	Negative	Mild positive	Negative	Positive

Optical clarity

**Figure 3:**
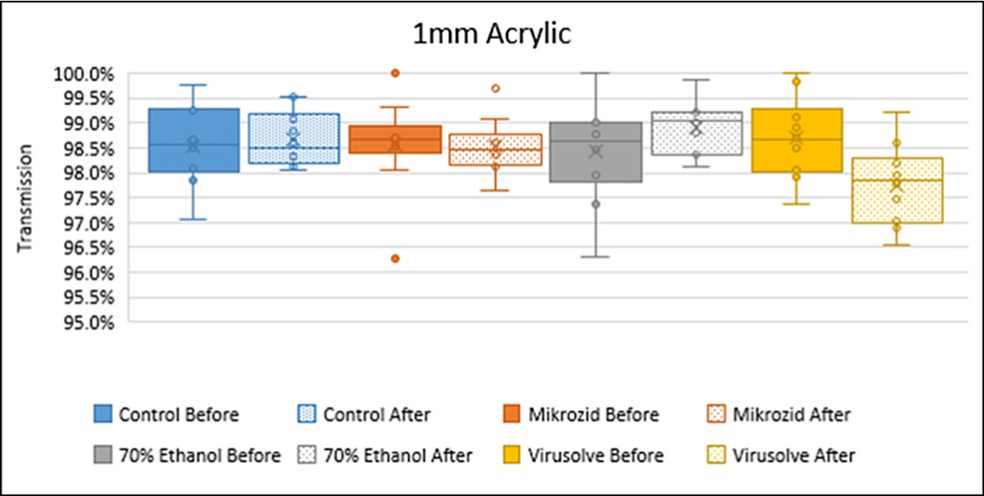
Optical clarity of the 1 mm acrylic after 200 cycles of repeated cleaning with commonly used hospital disinfectants

The optical clarity of the acrylic was measured via light transmission pre- and post-200 cycles cleaning. Using Student’s t-test, there was no significant change in the light transmission of the acrylic pre- and post-200 cycles of cleaning with 70% ethanol (98.6% and 98.5%, respectively) or with Mikrozid wipes (98.4% and 98.9%, respectively). With the use of Virusolve wipes, there was a small decrease in light transmission from 98.7% to 97.8%, p=0.02 (Figure 3).

COVID-19 viral antigen deposition

**Figure 4:**
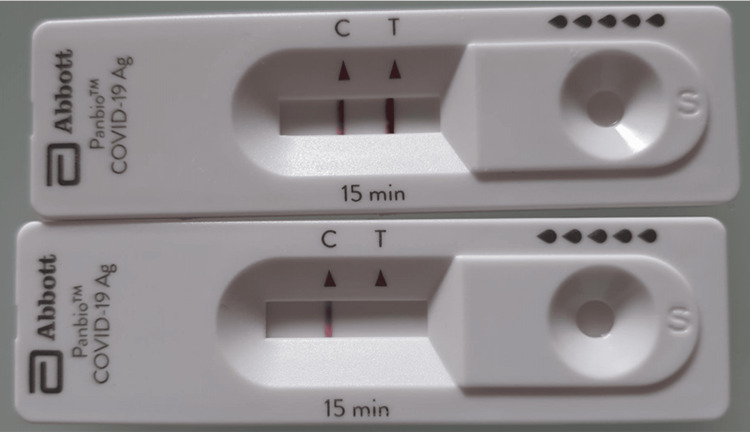
Covid-19 antigen lateral flow test results from a mid-turbinate swab (top) and the simultaneous swab of the proximal end of the suction tubing after 10 minutes of respiration

Five out of 20 study team members had tested positive for COVID-19 during the evaluation period. Four of these study members were fully vaccinated with Pfizer-BioNTech (COMIRNATY) mRNA COVID-19 vaccines and had subsequent Moderna (Spikevax) mRNA COVID-19 vaccine booster prior to COVID-19 infection. The fifth study member was fully vaccinated and had one subsequent booster with Pfizer-BioNTech (COMIRNATY) mRNA COVID-19 vaccines prior to infection. All five tubing inner lumens swabbed negative while the simultaneous mid-turbinate swabs demonstrated very strong positive test result (more intense than the control line) on the lateral flow test kits (Figure 4). 

## Discussion

While there are now several described barrier shields for use in anesthesia and radiology to protect frontline healthcare workers, many of these shields are big and bulky and were designed to be based in the parent anesthetic or radiology unit. While they could potentially be transported together with the ultrasound machine for bedside/intensive care ultrasound examinations, they would likely require a second operator to help with the transportation. Sir Flat CAP was primarily designed to be light and flat packed such that it may be easily transported by a single operator together with the ultrasound machine. A second consideration in the design of the SIR Flat CAP was the need to keep costs down by utilizing easily available materials. While it is possible to fabricate or purchase many of the previously described or commercially available barrier shields, the dedicated fabrication of available designs or commercially available units does not come cheap. By utilizing the shelf materials from hobby craft shops and other generally widely available hospital supplies, we hope that the design can be easily replicated worldwide and at a low cost in less well-resourced hospitals.

To the best of our knowledge, this is the first described supine cough droplet generation model for visualization of small droplet dispersion. Our supine cough generation model performed similarly to other previously published human supine cough models and, therefore, provides for a safe reproducible droplet generation in studies requiring simulated cough aerosolization in a supine position [10].

Our method of reverse fit test is the same test used for respirator fit testing for healthcare workers in our hospital as per infection control. For droplets too small to be visualized, reverse qualitative fit test results would suggest that the use of negative pressure suction with SIR Flat CAP results in a very effective reduction of invisible airborne transmission of microdroplets. Detection of fluorescence in the suction tubing also indicates that negative pressure contributes to the removal of microdroplets.

The use of hospital wall vacuums to provide the negative pressure within the confinement of the SIR Flat CAP would serve to provide a localized negative-pressure environment around the respiratory secretions of an infectious patient to protect frontline healthcare professionals in a manner similar to how negative-pressure rooms are used to isolate infective patients from the rest of the patients on the general ward. Usage of a small portable and reusable system would be of significantly lower cost than conversion of multi-bedded wards into negative-pressure isolation rooms. 

While it is potentially possible to use SIR Flat CAP without negative-pressure suction, the lack of air exchanges within the localized environment would reduce the efficacy of the barrier shield and would only provide the equivalent of a face shield to protect the healthcare professional from direct large droplet contamination. Smaller non-visible microdroplets may still escape through the gaps within the design into the environment surrounding the patient as evident by the faint positive noted by two of the nine assessors in the reverse qualitative fit test. A further concern with the use of Sir Flat CAP without negative-pressure suction raises concerns for potential carbon dioxide retention in the localized environment. With constant negative-pressure suction, fresh room air would be drawn in from the gaps between the barrier shield and the patient to the localized environment within, thus preventing the build-up of carbon dioxide and providing fresh air to the patient. Should oxygen be required, supplementary oxygen can still be supplied via nasal prongs or oxygen masks as required by the patient. Given that the maximal flow rate of hospital oxygen rarely exceeds 15 liters per minute and the minimal rating of hospital wall suction is 20 liters per minute via a Yankauer suction, adequate air exchange rates can still be achieved to protect the frontline healthcare worker. 

A further concern is that if SIR Flat CAP was used without suction, it would lead to the gradual accumulation of aerosolized viral particles within the confined space. This may raise the potential of increasing exposure to the healthcare worker if SIR Flat CAP was removed with a rapid motion allowing for the generated air turbulence to direct concentrated viral aerosol toward the healthcare worker. Using SIR Flat CAP with negative pressure would therefore be in keeping with the current recommendations from the United States Food and Drug Administration (U.S. FDA). The U.S. FDA previously alerted healthcare workers that the use of passive protective barrier enclosures without negative pressure may instead increase healthcare worker exposure to airborne particles in some circumstances [11].

While our experiments did not demonstrate any detectable COVID-19 antigen in the suction tubing, our current numbers are small with only five subjects who were fully vaccinated and boosted with mRNA vaccines and may not reflect the viral load seen in immunocompromised or unvaccinated patients. We would still recommend that the suction tubing be considered single-use only while the barrier shield itself could be easily cleaned and re-used.

The maintenance of high optical transmission and clarity despite 200 cycles of cleaning with standard hospital disinfectants supports the potential of extensive repeated use of the barrier shield with no significant compromise on optical clarity. High optical clarity in these small-volume barrier shields is important to reduce the sense of claustrophobia in the patients. 

While there have been many barrier shields described in use since the start of the COVID-19 pandemic, this is the first detailed practical evaluation of a low-cost, portable, negative-pressure barrier shield specifically designed for use by front line sonographers. While this is a limited proof of concept study dedicated toward use in COVID-19-positive patients requiring urgent portable ultrasound examinations, the results from this series of experiments could also be equally relevant to any other patients with airborne respiratory infections requiring respiratory precautions/isolation, such as tuberculosis or a future yet to be identified pandemic pathogen. 

Worldwide, hospitals are being overwhelmed and healthcare workers are resigning in record numbers. It is estimated that a healthcare worker is 11.61 times more likely to contract COVID-19 [12]. In an outbreak, confidence in safety and early training are some factors that reduce fear and promote healthcare workers' willingness to continue their work [13]. Fear and burnout can lead to maladaptive behaviors such as failure to report for duty, using scarce resources unnecessarily, or being less thorough in the assessment of a patient [14].

Many sonographers feel anxious working on the front lines of the pandemic [15]. Lack of a sense of psychological safety negatively impacts sonographers’ morale. There may also exist a subconscious tendency to rush the ultrasound procedure, with less time to optimize image quality and potential impairment of bedside interpretation. We hope that the introduction of this relatively low-cost and easily replicable SIR Flat CAP will help protect frontline sonographers and provide a sense of psychological security with resultant improvement in staff morale and examination quality.

As a low-cost and easily replicable product, there is a production limitation to the widespread adoption of SIR Flat CAP. Rapid scalability with buy-in from commercial developers is one of the biggest issues the team noted with regard to the design. The development team had previously engaged with several commercial plastic manufacturers to look into mass production of SIR Flat CAP but was unable to achieve success after an initial small-volume production trial run. As SIR Flat CAP was easily replicable, commercial entities were not keen to support production due to a lack of intellectual property protection due to the design. The ability to build and replicate SIR Flat CAP based on easily available common materials also limited the potential commercial value to the manufacturers. 

## Conclusions

In this paper, we describe an easily replicated, portable low-cost barrier shield to provide an additional layer of protection for front line sonographers against COVID-19. While the COVID-19 pandemic has been declared over, this barrier shield would still maintain relevance in protecting frontline healthcare workers from other airborne pathogens, especially in any future outbreak. To the best of our knowledge, this is the only described supine cough generation model that reliably produces similar visible droplets to published data of human supine cough droplet distribution. Using a combination of reverse qualitative fit testing with fluorescent droplet detection allows for evaluation and comparison of the effectiveness of various barrier shields at protecting frontline healthcare workers from visible and microdroplets.
